# Access to essential medicines and standard treatment for chronic diseases

**DOI:** 10.4103/0253-7613.66830

**Published:** 2010-06

**Authors:** Anita Kotwani

**Affiliations:** Department of Pharmacology, V. P. Chest Institute, University of Delhi, Delhi - 110 007, India. E-mail: anitakotwani@yahoo.com

Chronic diseases have emerged as a major killer globally. Nearly 60% of all deaths in the world are caused by chronic ailments such as heart disease, stroke, cancer, chronic respiratory diseases and diabetes. The World Health Organization (WHO) reports that of the 35 million (60%) people who died from chronic diseases in 2005, half were under 70, and half were women. Of these, 72% deaths were estimated to have occurred in developing countries, indicating an epidemiologic transition in these countries. In India, chronic diseases contribute to an estimated 53% of the deaths and 44% of disability-adjusted life-years lost.[1] A study conducted in 45 villages in East and West Godavari in Andhra Pradesh shows that noncommunicable diseases are a leading cause of death in rural India.[2] The implications of these findings are far-reaching.

Chronic diseases are a serious public health issue, particularly because they require long-term therapy. Management of chronic diseases has a complex and adverse impact on health systems in countries like India, which are already burdened with the unfinished agenda of communicable diseases as well as maternal and child health problems. Ensuring access to medicines for treating chronic diseases, however, remains neglected in most developing countries. Generally, the focus is on improving access to medicines for treating infectious diseases. For chronic diseases, prevention is the priority. However, it is well established that in addition to prevention, treatment of chronic diseases should form an essential component of any comprehensive public health programme.

Chronic diseases have a multidimensional impact on the community as well as on the individual, and require a lot of healthcare resources for their management. At the individual level, one faces financial problems, which is one of the most important reasons of non-compliance, resulting in complications. At the national level, chronic diseases have a huge economic impact. The WHO reports that India could forgo US $236 billion in national income over the next 10 years as a result of heart disease, stroke, cancer and diabetes.[3] Hence, this largely invisible epidemic of chronic diseases must be addressed through necessary actions by policy makers, regulators, doctors and researchers.

## Poor Access to Medicines

Available data point to a dismal scenario in India. Seven medicine price surveys were carried out (2003–4) in six states using a standard methodology developed by the WHO and the Health Action International to measure price, availability and affordability of a basket of essential medicines for acute and chronic diseases in the public and private sectors.[4,5] Availability of medicines was found to be very low in the public sector in all the states (median availability, 0–30%). The majority of the population purchased medicines from the private sector, where generics were usually available, although prices of certain medicines were high. Affordability is a big issue for most of our people. The World Bank reports (2006) that 34.7% of the Indian population earns < US $1 per day!

## Access to Essential Medicines for Bronchial Asthma

If we take bronchial asthma as an example of a chronic disease, we find that the situation is not encouraging. Data on the availability, price and affordability of two essential medicines for asthma, beclomethasone inhaler (50 *μ*g/dose) and salbutamol (0.1 mg/dose) inhaler, were collated from five price studies conducted in Haryana, Karnataka, Maharashtra, Rajasthan and Chennai.[6] Results revealed that no inhalers were on the procurement list or state essential medicine list, except Rajasthan. Hence, they were unavailable at any of the public facilities in Haryana, Karnataka, Maharashtra and Chennai.

In Rajasthan, both the inhalers were available only in the public facility of the capital city of Jaipur, but not in any other cities. This means that people living in parts of Rajasthan other than the capital city were not getting inhalers from the government-run facilities. Moreover, all citizens in the state are not entitled for free medicines in the public sector. Public facilities may be procuring inexpensive oral asthma medications, but those are not categorized as essential medicines as per the standard treatment guidelines. Patients are likely to experience asthma-related morbidity and mortality without essential inhalational asthma medicines. Poor patients have no other choice but to buy them from private facilities. Purchasing one inhaler each of salbutamol and corticosteroid costs around two days wages for the lowest-paid government worker.

A large proportion of the Indian population works in the unorganized sector and earns much less than the minimum wages prescribed by the government. Hence, affordability is a very important issue for the general population for treatment of any chronic disease requiring a life-long therapy. Medicines for any chronic disease should be dispensed for at least 1 month at all public facilities.

In India, many patients simply visit a retail pharmacy and purchase medicines without prescription. They rely on the advice of pharmacists. Usually, this practice is followed to save on time and expense of consulting a doctor. In Chennai city, when simulated clients with symptoms of mild persistent asthma visited private retail pharmacies, they were given oral bronchodilators, antibiotics, methylxanthines and oral corticosteroids rather than inhaled medications.[7] When poor patients visit a public facility, medicines are not available, and if they visit a retail pharmacy, they are not given the essential medicines. This implies that patients with financial constrains are not getting essential medicines for asthma.

Another Indian study shows that general practitioners (GPs) have a poor knowledge about prophylaxis for asthma. A majority of the GPs in Delhi (72%) reported salbutamol as the preferred prevention for asthma, while only 25% of the GPs considered inhaled medicines to be their preferred preventive treatment.[8] We have ourselves observed that in Delhi, of all the asthma patients visiting a public referral hospital for the first time, although most had already consulted more than two doctors, only 42% received an inhalation therapy and 4% were given a written treatment plan. At the same time, it is also observed that most patients consider asthma as an acute condition and very few take the inhalers regularly as prescribed. One of the important reasons for altering the dose or stopping the inhalation medication, apart from acute episodic belief of disease, is cost of therapy. Generally, patients and/or prescribers are treating acute episodes rather than focusing on long-term asthma control.

Treatment behavior for other chronic diseases like hypertension and diabetes is not likely to be different from bronchial asthma. For these chronic diseases, patients generally decrease the dose or stop the medication once they feel alright or if the relevant values come back in the normal range. The doctor–patient interaction is poor and no written treatment plan for chronic diseases is generally given. Studies should be carried out to determine the factors that are responsible at various levels for irrational treatment. Evidence-based intervention programmes should be implemented for the rational management of chronic diseases. Lessons can be learnt from developed countries, which will help in the generation of baseline data and, with the support of the international community, suitable interventions can be tested in low- and middle-income countries.[9] The government should fund more research studies on the rational use of medicines and on health policy that will eventually improve the national healthcare. Experience from western countries has demonstrated that huge gains can be achieved in prolonged lifetime productivity and well-being with modest investments in rational treatment of chronic diseases. Because the incidence of chronic diseases is increasing rapidly in developing countries like India, there is an urgent need for improving access to essential medicines, treatment guidelines, education and awareness for doctors and general public, policy making and resource allocation for such diseases.
